# Three-Dimensional Printing: is it useful for Cardiac Surgery?

**DOI:** 10.21470/1678-9741-2019-0475

**Published:** 2020

**Authors:** Marcos Aurélio Barboza de Oliveira, Carlos Alberto dos Santos, Antônio Carlos Brandi, Paulo Henrique Husseini Botelho, Domingo Marcolino Braile

**Affiliations:** 1 Department of Cardiac Surgery, Hospital Santo Antônio and Femina Cuiabá, Sinop, Mato Grosso, Brazil.; 2 Department of Cardiovascular Surgery, Universidade Federal do Mato Grosso, Sinop, Mato Grosso, Brazil.; 3 Hospital de Base, São José do Rio Preto, São Paulo, Brazil.

**Keywords:** Imaging, Three-Dimensional, Printing, Three-Dimensional, Cardiac Surgical Procedures, Surgeons, Heart

## Abstract

**Introduction:**

The medical use of three-dimensional (3-D) images has been a topic in the literature since 1988, but 95% of papers on 3-D printing were published in the last six years. The increase in publications is the result of advances in 3-D printing methods, as well as of the increasing availability of these machines in different hospitals. This paper sought to review the literature on 3-D printing and to discuss thoughtful ideas regarding benefits and challenges to its incorporation into cardiothoracic surgeons’ routines.

**Methods:**

A comprehensive and systematic search of the literature was performed in PubMed and included material published as of March 2020.

**Results:**

Using this search strategy, 9,253 publications on 3-D printing and 497 on “heart” 3-D printing were retrieved.

**Conclusion:**

3-D printed models are already helping surgeons to plan their surgeries, helping patients and their families to understand complex anatomy, helping fellows and residents to practice surgery, even for rare cases, and helping nurses and other health care staff to better understand some conditions, such as heart diseases.

**Table:** 

Abbreviations, acronyms & symbols	
2-D	= Two-dimensional
3-D	= Three-dimensional
CHD	= Congenital heart disease
CNC	= Computer numerical control
CT	= Computed tomography
DORV	= Double outlet right ventricle
FFF	= Fused fiber filament
STL	= Stereolithographic
VSD	= Ventricular septal defect

## INTRODUCTION

Advanced two‐dimensional (2-D) technology has evolved into three‐dimensional (3-D) imaging, which is now powerful and has begun to transform the field of medicine^[1]^. These 3-D images provide more information than flat 2-D images produced by ordinary X-rays, echocardiograms, and even computed tomography (CT) scans, all of which are often unable to provide surgeons with complex anatomical details^[1,2]^.

There is an increasing number of computer programs (such as Materialise Mimics™) that combine all of the 2-D images produced by ultrasounds, echocardiography, CT-scans, and magnetic resonance imaging to create complete and reliable 3-D models that can be spun, virtually cropped, and zoomed in or out in order to help surgeons to establish better preoperative plans^[2,3]^. Despite the benefits of these 3-D images, however, they are essentially still 2-D images of 3-D models on a canvas.

The newest technology can create (or “print”) a physical object based on the 3-D model. In this way, the model can be touched and physically manipulated, and it is useful in its similarity to the true anatomy in question. These 3-D objects are helping an increasing number of doctors, doctors-in-training, and other health care professionals around the world to plan surgeries, study complex anatomy, and explain pathology and surgeries to patients and their relatives^[2,4]^.

This article will describe types of 3-D printers, the materials used to print 3-D objects, and the uses of 3-D printing in adult and pediatric cardiac surgery.

## METHODS

### Searching the Database

**Fig. 1:**
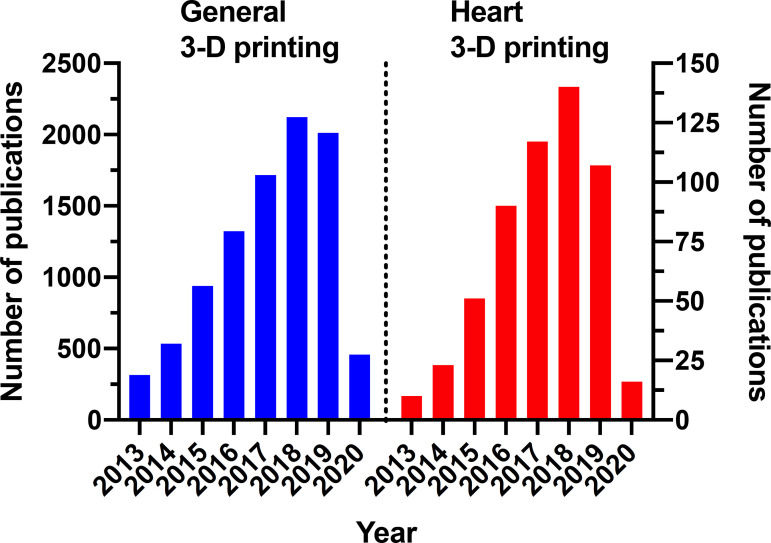
The number of publications from 2013 to 2020 divided into “general” and “heart” categories. Note that the proportion between the groups is constant year by year.

A comprehensive and systematic search of the literature was performed in PubMed and included material published as of March 2020. No constraints were applied to the publication date in order to perform a search as comprehensive as possible. Keywords included “printing, three-dimensional” [MeSH Terms] OR (“printing” [All Fields] AND “three-dimensional” [All Fields]) OR “three-dimensional printing” [All Fields] OR (“3d” [All Fields] AND “printer” [All Fields]) OR “3d printer” [All Fields] or “rapid prototyping”. Using this search strategy, 9,253 publications on 3-D printing were retrieved. Interestingly, the first paper was published in 1988^[5]^, but 95% of the articles were published after 2013, or in the last six years. When the search was further refined to include the word “heart”, the number of publications decreased to 497, or 5% of total publications. A more detailed analysis is provided in Figure 1.

## RESULTS

### Methods for 3-D Printing

3-D printers vary in their scope and properties and range in price over several orders of magnitude. An understanding of the advantages and limitations of each 3-D printer method is helpful, and the ideal selection is contingent upon the application.

#### Material Extrusion (Fused Fiber Filament [FFF])

The most common type of 3-D printers, fused deposition modeling printers, use material extrusion technology. They create 3-D objects by passing a plastic wire into a heated extruder (a nozzle) and liquifying it. In this way, the object is built up layer by layer by laying down thin strips of the liquefied plastic material. As each layer is printed, either the print bed or the nozzle moves to allow a second layer to be built upon the first. This method of printing is widely available, and the printers are relatively cheap. The material used is generally low cost and readily available. The print quality is dependent upon the exact printer but it can be quite high, with resolutions in the order of 0.2 mm^[3]^.

**Fig. 2:**
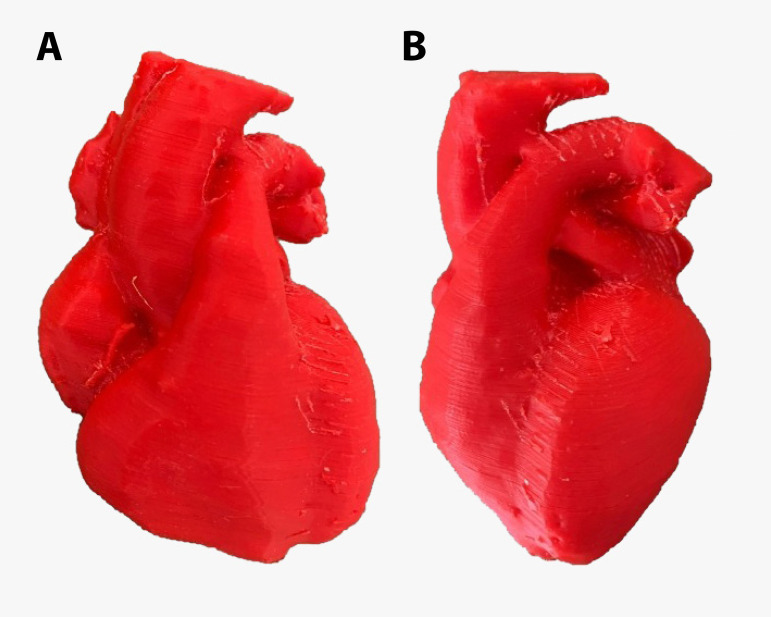
An example of a fused deposition modeling object. A) Anterior view of a model of the heart. B) Lateral view of a model of the heart.

The FFF 3-D printer can print complex objects, such as the heart represented in Figure 2. In this case, the material used was plastic filament, the shell thickness was 1 mm, the infill was 15%, grid support material was used only on the bottom, the head temperature used was 240 ˚C, and the velocity was 4800 mm/min. This heart took 20 hours to be printed.

#### Vat Polymerization

In this method, printers rely on a directed light source to excite a photopolymer. Using a projector or laser, each layer of material is polymerized and attached to a print bed to build up multiple layers. Vat polymerization printers are used in the jewelry industry, but they may also be useful for printing coronary arteries or valve tissue. They are modestly priced and easily available. The cost of the resin is higher than that of the plastic used in material extrusion printers^[3]^.

#### Powder Bed Fusion

Powder bed fusion printers use a laser to heat and fuse a bed of powdered printing material such as metal or nylon. The laser melts and binds each layer of the 3-D object together. After a layer is printed, rollers place a small layer of powder above the printed layer, which is then fused with the underlying layer of material. Multiple layers are combined to form a 3-D printed object. Nylon is often used as a low-cost and robust material that may be sterilizable. Other materials, including titanium, have been used for printing prostheses, such as bone implants. The printers required for this are currently quite expensive, though the material costs are low when objects are printed in large batches^[3]^.

#### Binder Jetting

Printers that utilize binder jetting technology use a binder, such as glue, to combine layers of fine material, such as finely ground plaster or plastic particulates, layer by layer. After one layer of the object has been bound, another layer of powder is added to the bed, and the process is repeated until the 3-D model is printed. Most traditional multi-color inkjet printers use a binder jetting technique. The outer layer or visible portion of each slice in the model can be colored using a method similar to that of inkjet printing after the binding material has been jetted onto the powder. These models may be visually attractive and can be used to represent different anatomical structures. Binder jetting printers are generally modestly expensive, and printed parts all need to be vacuumed or cleaned at the time of printing, typically in a work station specifically dedicated to this task, in order to avoid the spread of fine dust particles^[3]^.

#### Material Jetting

This method involves multiple small jets laying down a photopolymer above an underlying layer. These jets may lay down various forms of plastic or rubber-like materials in multiple colors. They may also lay down a support material. Each layer is exposed to ultraviolet light, which hardens the liquid material. Using this method, multiple layers are built up to form the 3-D model. Soluble material, which may be meltable wax or water-soluble photopolymer, is removed to reveal the final object. These printers are generally more expensive than those described previously but may allow for the printing of an object with multiple types of materials. The materials’ properties may also be altered, for example, to add flexibility to certain areas of the object, which, in the field of medicine, can result in a more anatomically correct representation of an organ^[3]^.

#### Computer Numerical Control (CNC) Routers

CNC routers provide another method to create a 3-D object. While 3-D printing produces an object by adding layers of material, CNC routers work by sculpting the image from a solid piece of material. CNC router machines rely on a drill mounted on a robotic arm linked to a computer. The computer has a specific program to transform a stereolithographic (.STL) file into very specific moves to erode the material without breaking it. Different kinds of material, such as wood, polyvinyl chloride, aluminum, steel, or titanium, may be used in CNC routers. Each material has its own bit. Our search found no papers in the literature on the creation of customizable prostheses using these machines, but this application seems plausible. The technology is used in cardiovascular surgery to make molds to inject plastic to venous reservoir, oxygenators, and coverage for filters in extracorporeal circulation.

### 3-D Models in Adult Cardiac Operations

Benke et al.^[6]^ published a case report on a patient with a pseudoaneurysm followed by aortic valve replacement and a modified Bentall-Debono procedure in which the preoperative 3-D printed model was the cornerstone of the surgical planning process. The pseudoaneurysm was very close to the ribcage and sternum, and the opening of the sternum would risk the explosion of the pseudoaneurysm. The model allowed the surgeons to visualize all anatomic relations between the heart, aorta, pseudoaneurysm, and sternum in the printed model, helping them to decide to put the patient under hypothermia prior to opening the sternum. The pseudoaneurysm ruptured during surgery, but the patient was already under hypothermia, and the surgeon was able to repair the lesion. The patient was discharged 12 days after surgery.

In another case, Hermsen et al.^[7]^ printed a 3-D model of a heart with hypertrophic cardiomyopathy. In this case, the surgeons operated first on the model, addressing every anatomic difficulty and considering the maximum depth of each incision at which ventricular septal defect could be avoided. They also used another kind of filament - hydrogel - which more closely mimics heart tissue. They concluded that “operative rehearsals with patient-specific 3-D prints may reveal to a surgeon potential pitfalls, a preferred approach, or optimal instrumentation, at the same time providing a preview of anatomic nuance and potentially forming muscle memory.”.

### 3-D Models in Pediatric Cardiac Operations

Congenital heart disease (CHD) affects a large number of children every year. The incidence of CHD is generally considered to be eight per 1,000 live births^[8]^. Perhaps more challenging than determining the true incidence of CHD is addressing the unique nature of each case and determining the most efficient ways to train surgeons and plan each surgery. One complicating factor is that each patient’s anatomy can change the choice of surgical method, but this anatomy may not be fully understood until the time of surgery^[9,10]^.

In their study, Zhao et al.^[11]^ enrolled 25 children (eight patients in the 3-D model group and 17 patients in the control group) with double outlet right ventricle (DORV) in order to assess the role of 3-D printed models in preoperative planning strategies for the surgical repair of DORV. Though a small number of patients were enrolled, the team found a significant difference in mechanical ventilation time and intensive care unit stay, as well as a marginal significance (P=0.09) in aortic cross-clamp time. This shorter time in the 3-D group undoubtedly led to better outcomes. They concluded that 3-D printing has the potential to improve surgical efficiency and postoperative outcomes.

Biglino et al.^[12]^ report a case of an 11-year-old male patient with repaired truncus arteriosus and right pulmonary artery stenosis. The 3-D model helped the surgeons to determine the best place to divide the aorta to reach the right branch behind it. In some similar cases, a 3-D model would not be necessary, but the fact that this was a reoperation and that all of the structures were very tight meant that knowledge of the complete anatomy prior to surgery made the procedure much faster and safer for the patient. A tangible 1:1 scale model was also found to be much more useful for the surgeon than an image on a screen.

### 3-D Models in Professional Training

3-D models are revolutionizing how residents^[13]^ and nurses^[14]^ are trained. Health care professionals can learn from models based on real cases, and students may draw on, cut, and even operate on 3-D models. If models are damaged or destroyed, new ones can be produced. What’s more, attending physicians and professors have more options for course planning and don’t need to wait for a patient to present with a very rare congenital condition in order to teach students about it^[15,16]^.

In small hospitals and residency programs, residents may not have the opportunity to see all types of CHDs by the end of their training, but this problem can be reduced using 3-D models. To this end, 3-D models can be anonymized and shared among training centers, allowing more institutions to have access to a large library of different types of CHDs, and to print 3-D models on demand. Digital libraries can also allow the same .STL file to be printed with transparent, colored, or malleable materials so that models may be used for different purposes. Examples include the use of 3-D models to show surfaces or inner structures, or to practice cutting structures with a scalpel, as in a real surgery^[7,15]^.

### 3-D Models for Patient and Family Education

Biglino et al.^[15]^ assessed the usefulness of 3-D models with patients and their families. According to the authors, patients liked to see and touch the models because they “help visualize what’s going on inside”. The authors found that patients were more actively involved in the conversation, without showing any level of anxiety or discomfort in discussing their cases with the models in their hands.

The same group of authors also evaluated the parents of the previously described children. They found that all parents agreed that the models stimulated curiosity and allowed them to engage the children in their treatment. Also, the parents also preferred patient-specific models over lesion-specific models^[15]^.

## DISCUSSION

3-D printing is changing the way we plan surgeries, the way we discuss the case among all health care staff (surgeons, cardiologists, nurses, physiotherapists, practitioners), the way we teach residents, fellows, and students, and how we discuss the problem and the surgery with patients and their relatives. The didactic benefits of 3-D printing are summarized in Table 1. Lau et al.^[17]^ assessed the intentions of surgeons and cardiologists using 3-D models in communications within their medical practices. Overall, reports were positive regarding the use of the models in their offices, but some of the professionals complained about the long duration of the consultation. When they used the 3-D model, the average consultation time increased by five minutes. Some may argue that this increased consult time reflects more in-depth discussions about cases with parents and patients, a change which may have positive consequences. Another reason for this longer consultation is that most of the doctors surveyed reported using the model in addition to 2-D images provided from scans, such as ultrasound and CT. This dynamic may indicate that 3-D printed models are not able to replace existing approaches, but instead serve as a complementary tool for communication between doctors and patients. This choice could also be the result of physicians’ discomfort with using the 3-D model alone to discuss cases with their patients. If the latter possibility is, in fact, true, the need for both types of materials will soon be replaced by the exclusive use of 3-D models once their use is normalized for doctors and patients alike.

**Table 1:** Applications of three-dimensional (3-D) printed modeling to cardiovascular diseases.

First author	Clinical condition	Application of 3-D printed model
Benke K^[6]^	Aortic pseudoaneurysm	Used to guide intervention approach
Deferm S^[1]^	Tetralogy of Fallot	Helped the surgeon to locate the aortopulmonary collateral arteries
Biblino G^[12]^	Truncus arteriosus	Showed the anatomy to surgeons, trainees, and relatives
Yoo SJ^[16]^	CHD in general	Pre-interventional procedural practice, enabled real surgery on the model
Jonas RA^[13]^	CHD in general	Training fellows
Zhao L^[11]^	Double outlet right ventricular surgery	Helped the surgeon to know the VSD boundaries
Olejnik P^[9]^	CHD in general	Preoperative planning
Hermsen JL^[7]^	Hypertrophic cardiomyopathy	Showed the anatomy, enabled real surgery on the model

CHD=congenital heart disease; VSD=ventricular septal defect

We believe 3-D printing will soon be incorporated in the routines of large medical centers, because the fast increase in the number of published papers reflects a parallel increase in interest among the scientific community, and the increase in the number of different authors writing about the same matter reflects an increase in the number of professionals testing out this new option. This trend doesn’t seem to be temporary, a likelihood which is confirmed by literature: the consistent yearly increase in publications reflects the solid upward trends in 3-D printing use and availability, and this technique will likely thrive as more centers make it available.

The speed of the increase in the number of centers using 3-D printing is inversely related to the price of the technique. The calculations used to make this determination consider the costs of the 3-D printer, the supplies (plastic, nylon, metal, to name some), the software used to render the 3-D image, the professionals required to operate the hardware and software, and doctors trained to select the cases. Over the years, however, 3-D printing has become more cost-effective, and this barrier is being lowered^[3,4]^.

The medical industry is just now scratching the surface of 3-D printing potential. Future possibilities include smaller printers, cheaper printers, 3-D printers for home use, and, in the medical field, perhaps even printers capable of making compatible and fully functional organs. This technology therefore has the potential to revolutionize organ transplant. 3-D printers may one day make it possible for patients with chronic kidney disease, heart failure, or liver failure to receive an organ transplant without the need to wait for a match from a donor^[4]^.

## CONCLUSION

Three-D printed models are already helping surgeons to plan their surgeries, helping patients and their families to understand complex anatomy, helping fellows and residents to practice surgery, even for rare cases, and helping nurses and other health care staff to better understand some conditions, such as heart diseases.

**Table:** 

Authors' roles & responsibilities	
MABO	Conception and design of the work; acquisition, analysis, interpretation of papers for the work; drafting the work and revising it critically for important intellectual content; final approval of the version to be published
CAS	Interpretation of papers for the work; final approval of the version to be published
ACB	Interpretation of papers for the work; final approval of the version to be published
PHHB	Interpretation of papers for the work; final approval of the version to be published
DMB	Conception and design of the work; acquisition, analysis, interpretation of papers for the work; drafting the work and revising it critically for important intellectual content; final approval of the version to be published
